# Application of *Saccharomyces cerevisiae* var. *boulardii* for Biological Detoxification of Chemical Contaminants in Foods: A Comprehensive Review

**DOI:** 10.3390/foods14244260

**Published:** 2025-12-10

**Authors:** Karina Nascimento Pereira, Amanda Cristina Dias de Oliveira, Handray Fernandes de Souza, Sana Ullah, Usama Nasir, Sher Ali, Carlos Augusto Fernandes de Oliveira

**Affiliations:** 1 Department of Food Engineering, School of Animal Science and Food Engineering, University of São Paulo, Av. Duque de Caxias Norte, 225, Pirassununga 13635-900, SP, Brazil; 2 Department of Food Science & Technology, Faculty of Food Science and Nutrition, Bahauddin Zakariya University, Multan 60000, Pakistan

**Keywords:** adsorption, probiotic yeast, toxicity, detoxification

## Abstract

The global food supply is increasingly challenged by toxicologically relevant natural and synthetic chemicals, including mycotoxins, pesticides, heavy metals, and migrants from food packaging. Conventional physical and chemical detoxification approaches can reduce contaminant loads but may compromise nutritional and sensory quality or leave residues, motivating a shift toward biological strategies. This review synthesizes current evidence on *Saccharomyces cerevisiae* var. *boulardii*, a clinically established probiotic yeast, as a multifaceted biological detoxification agent in foods. We outline its dual modes of action: (i) rapid, reversible adsorption of contaminants mediated by the architecture of the yeast cell wall (β glucans, mannans, chitin), and (ii) active biotransformation through secreted proteins and enzymes. *S. cerevisiae* var. *boulardii* has been reported to remove up to 96.9% of aflatoxin M_1_ in reconstituted milk, depending on strain, dose, contact time, pH, and matrix effects. We collate findings for other contaminant classes and highlight practical variables that govern efficacy, while comparing detoxification performance with bacterial probiotics and conventional methods. Critical knowledge gaps were highlighted, including standardized testing protocols, mechanistic resolution of adsorption versus degradation, stability and regeneration of binding capacity, sensory impacts, with scale up and regulatory pathways. A roadmap is proposed to harmonize methods and unlock the full potential of this promising biotherapeutic yeast for food safety applications.

## 1. Introduction

Foodborne chemical contaminants from environmental sources, processing operations, and packaging materials pose continuing risks to consumer health [1,2,3]. Their adverse effects including oxidative stress, inflammation, metabolic dysfunction, and intestinal disturbances underscore the need for effective mitigation strategies [4,5]. Although physical and chemical detoxification methods are widely used, these approaches may compromise nutritional or sensory quality or leave undesirable residues [6,7]. To address these limitations, interest has grown in biological strategies employing probiotic microorganisms as safer and more sustainable alternatives in food systems [5,8,9]. When administered in adequate amounts, they benefit health to support microbiota homeostasis, strengthen intestinal barrier, and reduce pro-inflammatory cytokines [10,11,12]. Recent research has increasingly emphasized the capacity of specific microorganisms, particularly yeasts, to mitigate foodborne contaminants. Several strains reduce chemical contaminants through adsorption to cell-wall components or through enzymatic biotransformation, with efficacy depending on the contaminant type, strain characteristics, and food matrix conditions [1,13,14,15]. For example, lactic acid bacteria (LAB) and *Saccharomyces cerevisiae* show strong adsorption of aflatoxin M_1_ (AFM_1_) in ultra-high temperature (UHT) milk [16], and patulin removal by *S. cerevisiae* depends on cell-wall β-1,3-glucan composition [17]. A LAB–yeast consortium (*Lactobacillus rhamnosus* + *S. cerevisiae*) has achieved up to 98.4% AFM_1_ detoxification in milk [18].

Within this context, *Saccharomyces cerevisiae* var. *boulardii* has emerged as a uniquely promising probiotic yeast due to its dual relevance in human health and food applications. While bacteria such as *Lactobacillus acidophilus*, *Lacticaseibacillus casei*, and species of *Bifidobacterium* (e.g., *B. bifidum*, *B. lactis*, *B. longum*, *B. thermophilum*) remain dominant in commercial probiotics [12,19,20], *S. cerevisiae* var. *boulardii* stands out for its robust survival, therapeutic properties, and detoxification potential. It has been effectively used to manage intestinal diseases [20], demonstrates high cell viability in fermented beverages [21], and exhibits notable capacity to reduce contaminants in dairy matrices [22,23]. *S. cerevisiae* var. *boulardii* was first isolated from lychee and mangosteen fruit peels by Henri Boulard, who observed that traditional fruit infusions alleviated cholera symptoms during an outbreak [24]. Since then, *S. cerevisiae* var. *boulardii* has been widely used in the treatment of intestinal diseases and is considered one of the main probiotic microorganisms in the pharmaceutical and food industry [24,25]. Despite being taxonomically classified within *S. cerevisiae*, it differs markedly in physiology and stress tolerance. Its optimal growth at 37 °C, enhanced acid resistance, and distinct metabolic traits set it apart from conventional *S. cerevisiae* strains and reinforce its efficiency as both a probiotic and a decontaminant [26,27]. Table 1 summarizes the distinguishing features supporting its unique functional role.

Critical physiological and genetic differences between *S. cerevisiae* var. *boulardii* and *S. cerevisiae* strains (Table 1) help explain its superior performance as a probiotic and its emerging role in contaminant detoxification. *S. cerevisiae* var. *boulardii* exhibits optimal growth at 37 °C and shows enhanced survival under simulated gastric conditions (pH ≤ 2) and in the presence of bile salts, supporting its persistence during gastrointestinal transit and in acidic food matrices. Additional copies of *FLO* genes and associated flocculation capacity promote adhesion to microbial cells and contaminants, improving toxin binding and removal. Variations in cell-wall composition and stress-response gene dosage further contribute to environmental resilience and influence adsorption behavior. The lack of sporulation reduces spoilage risks in food applications, while differences in carbohydrate utilization including the inability to metabolize galactose remain relevant for selecting strains for specific fermentation processes [28,29,30].

To confer probiotic benefits, microorganisms must survive gastrointestinal transit and act beneficially within the host. *S. cerevisiae* var. *boulardii* influences multiple intestinal processes. It exerts anti-inflammatory activity by promoting anti-inflammatory cytokines and stimulating immunoglobulin A production. It also competes with pathogens for receptor binding sites, suppresses virulence traits, maintains epithelial integrity, and inhibits *Candida albicans* filamentation and biofilm formation [31,32]. Furthermore, it reduces adherence and invasion by *Salmonella enterica* serovar Typhimurium and enteropathogenic *Escherichia coli* (EPEC), protecting tight-junction proteins [26,33,34]. As a non-colonizing, transient yeast, *S. cerevisiae* var. *boulardii* competes effectively for nutrients and adhesion sites, promoting the removal of pathogens through its expanded flocculin repertoire [35,36,37]. These mechanisms complement adsorption and biotransformation processes, expanding its relevance to contaminant detoxification. *S. cerevisiae* var. *boulardii* also secretes digestive enzymes and proteins capable of binding toxins and modulates the synthesis of short- and branched-chain fatty acids [24,37,38,39,40,41]. Given its distinctive physiological attributes and growing relevance in food processing, a focused assessment of its roles in detoxifying chemical contaminants is warranted.

Accordingly, this review synthesizes current knowledge on the capacity of *S. cerevisiae* var. *boulardii* to bind, transform, and mitigate foodborne chemical contaminants. We examine its biological mechanisms, summarize evidence across contaminant classes and food matrices or study type (in vitro/animal), and highlight key research gaps and future directions for translating this yeast into practical food-industry applications.

## 2. Overview of Food Contaminants Assessed for Detoxification by *S. cerevisiae* var. *boulardii*

**Figure 1:**
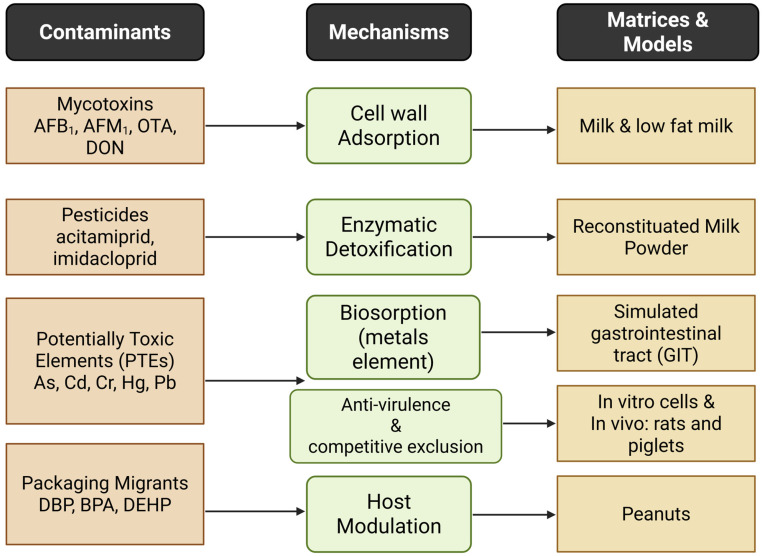
Major contaminant types with the detoxification mechanisms in the commonly used food matrices or experimental models.

Figure 1 provides a visual overview linking the contaminant classes covered in this study to the major detoxification mechanisms and the matrices/models most frequently investigated.

### 2.1. Mycotoxins

Several common and toxicologically relevant food contaminants occur in nature. Among them, mycotoxins are toxic compounds originating from the secondary metabolism of some fungal species such as *Aspergillus*, *Penicillium*, *Fusarium*, and *Alternaria*. In terms of occurrence in food, the main ones responsible for toxicity include aflatoxins (AF), ochratoxin A (OTA), patulin (PAT), fumonisins (FBs), deoxynivalenol (DON), zearalenone (ZEN), and others [42]. The production of these mycotoxins can occur at different stages of the food production chain; however, they are most frequently found under field conditions or during improper storage of grains and feed [9]. The United Nations Food and Agriculture Organization (FAO) estimates that mycotoxins contaminate more than 25% of the world’s cereal crops, with some reports suggesting this figure could be as high as 60% to 80% [43]. Mycotoxins can be found naturally in a variety of food products, including fruits, rice, beans, corn, peanuts, wheat, barley, spices, and milk, among others, causing significant damage to crops [44,45]. The health and economic implications of this contamination are profound [46]; as a consequence, humans and animals are directly exposed to mycotoxin toxicity upon consumption of contaminated food. The toxic effects depend mainly on the type, concentration, and exposure time of each mycotoxin. Most of these contaminants can cause hepatotoxicity, gastrointestinal toxicity, neurotoxicity, immunosuppression, and carcinogenicity and are therefore considered a global public health problem [45,47]. The International Agency for Research on Cancer (IARC) has categorized AFB_1_ as a Group 1 human carcinogen, while FB_s_ and OTA are classified as Group 2B human carcinogens [48]. Furthermore, the prevalence of masked mycotoxins that evade conventional detection methodologies is an emerging concern in the field of food safety.

### 2.2. Pesticides

Insecticides are a type of pesticide used to control pests in crops and restrict the invasion of insects, weeds, and others that adversely affect plant growth. Pesticides are primarily used to enhance crop production and prevent disease; however, their widespread use makes them one of the major chemical contaminants to which living beings are exposed [49]. The transfer of certain pesticide classes, such as neonicotinoids, to food indicates their potential to harm human health due to their systemic ability to penetrate plant tissues and accumulate in edible parts [50,51]. Diseases such as Parkinson’s, Alzheimer’s, reproductive disorders, chronic respiratory diseases, and different types of cancer are associated with exposure to pesticides [52]. The use of pesticides in agricultural production is essential to meet industrial needs and global food security, but their use also promotes high health risks for the environment and living beings [49]. The acute toxicity caused by pesticides is associated with their inhalation, ingestion, and direct contact with eyes or skin. In contrast, long-term exposure can cause chronic toxicity such as neurotoxicity, mutagenicity, carcinogenicity, and endocrine disturbance [53].

### 2.3. Packaging Migrants (Phthalates and Bisphenol A)

Phthalates and bisphenol A are chemicals widely used in the manufacture of plastics, ranging from beverage and food containers to medical devices [54]. Food is the most common form of exposure to these constituents due to their potential to leach into food. Leaching from plastic materials is facilitated by several degradation processes, including chemical, physical, biological, or photodegradation. Factors including heat and ultraviolet exposure, mechanical stress, or microbial processes weaken the polymer structure, allowing these chemicals to migrate into the surroundings [55]. These chemicals are considered endocrine disruptors, as they can interfere with hormone function and also cause developmental and reproductive disorders [56]. The harmful effects of these contaminants are due to their high lipophilicity, which facilitates absorption by different routes and accumulation in tissues [57]. Chronic exposure to these chemical components has been associated with adverse health effects, including developmental issues from pregnancy to adulthood, alterations in the nervous system and brain, and increased vulnerability to breast and liver cancer [58].

### 2.4. Potentially Toxic Elements

Potentially toxic elements (PTE), another class of non-biodegradable pollutants, also pose a significant health risk. They are naturally present in the earth’s crust, but with increasing urbanization and industrialization, the probability of their accumulation in the environment has increased extensively [59]. Consequently, human exposure to PTE occurs through several routes, including inhalation, ingestion, and skin contact, as well as direct consumption of crops irrigated with contaminated water [60]. Similarly, plants readily absorb PTE, which can bioaccumulate in their tissues and be transferred through the food web, increasing exposure to humans and animals [51,61]. This exposure is alarming due to their persistent, nondegradable, and toxic nature, even at low concentrations. Excessive involuntary exposure to PTE in the food chain is due to the presence of these compounds in water, air, and soil. The high toxicity of these PTE, even at low concentrations, makes them a major threat to food safety and human health [62]. PTE such as mercury, lead, chromium, cadmium, and arsenic cause serious damage to public health. Depending on exposure time, individual sensitivity, and metal type, exposure is linked to several harmful consequences, including immune system dysfunction, neurological diseases, skin lesions, liver disease, and kidney cancer [63,64].

## 3. Detoxification of Food Contaminants by *S. cerevisiae* var. *boulardii*

Chemical food contamination leads to significant food losses, reduces the value of food products, and poses substantial risks to human health [65]. The widespread and persistent nature of these contaminants, coupled with the limitations of existing physical and chemical detoxification methods, emphasize a substantial need for a new, more sustainable approach to food safety. The use of microorganisms to detoxify xenobiotics in food has been highlighted, and probiotic bacteria and yeasts have been shown to be effective in their ability to bind chemical compounds [14]. It has also been shown that probiotics during intestinal transit can inhibit absorption, leading to a decrease in the availability of toxic compounds to the body. Likewise, they can decrease the poisonous capacity of these compounds through binding or altering their structural integrity and facilitate their excretion [1,66,67]. Yeasts adsorb toxic compounds through the surface of their cell walls, and both viable and non-viable cells have a high capacity for reducing toxins in food and feed. The different compositions of the yeast cell wall give rise to considerable variation in their ability to adsorb toxic components [13,68]. In bacteria, the cell wall structure is composed of proteins, teichoic acids, peptidoglycans, and polysaccharides, which serve as binding sites. In the presence of chemical compounds that have negatively charged groups such as carboxyl, hydroxyl, and phosphoryl, they are able to bind to the bacterial surface [14,17,69]. Rapid adsorption via mannans/β-glucans/chitin is a shared feature of *Saccharomyces* spp., and is therefore not unique to *S. cerevisiae* var. boulardii. Reported differences in β-glucan content, mannoprotein patterns, surface charge, and flocculation phenotypes can, however, create strain-specific performance alterations across *S. cerevisiae* and *S. boulardii* [70,71]. Compared with LAB, which possess peptidoglycan/teichoic-acid-rich cell walls and acidify the medium, *S. cerevisiae* var. bouvardias offers robust tolerance to low pH and bile, and sustained viability at ~37 °C, making it suitable for co-ingestion scenarios [72]. For pre-consumption applications in foods where yeast growth is undesirable (e.g., fresh dairy), heat-inactivated cells or purified cell-wall fractions of *S. cerevisiae* var. *boulardii* and/or LAB adjuncts may be preferable. Organism choice should be guided by contaminant class, matrix, and process constraints.

### 3.1. Mechanisms of Detoxification

*S. cerevisiae* var. *boulardii* counters toxins through several biochemical routes. Initially, *S. cerevisiae* var. *boulardii* secretes a serine protease that cleaves toxin molecules and prevents their engagement with epithelial receptors, thereby neutralizing their effects [37,73]. It also modulates host immunity by dampening toxin triggered inflammatory signaling cascades and by promoting production of specific anti toxin IgA [74,75]. Under certain conditions, the yeast produces acetic acid, lowering gut pH and inhibiting many harmful microorganisms and their toxins [37,72]. The secreted protease, together with other factors, can bind toxins or alter the epithelial surface so that receptor binding is hindered [76,77]. By blunting toxin action and inflammation, *S. cerevisiae* var. *boulardii* helps preserve tight junctions and overall barrier integrity, reducing fluid leakage into the intestinal lumen [77,78]. In addition, it may directly interfere with pathogen physiology by competing for substrates, modifying the local environment, or perturbing bacterial processes [75].

**Figure 2:**
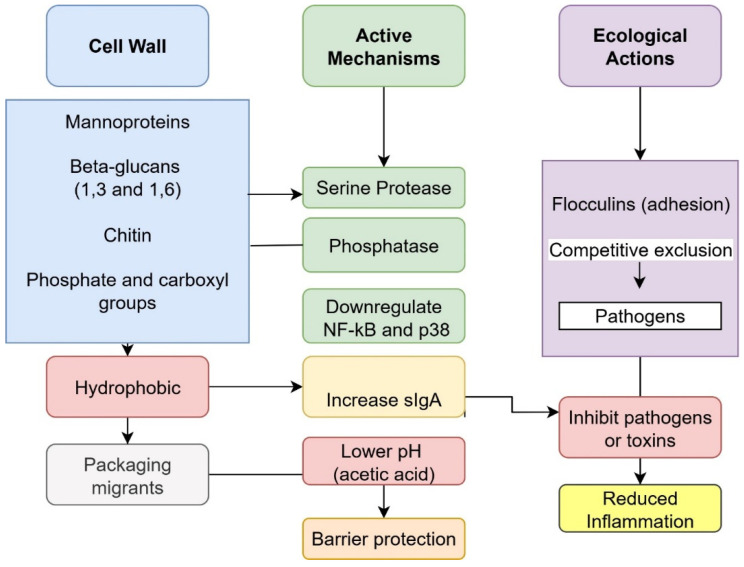
Mechanisms of *Saccharomyces cerevisiae* var. *boulardii* in food detoxification.

*S. cerevisiae* var. *boulardii* acts through more than one route in food detoxification, combining passive binding with enzymatic activity and pathogen antagonism [29]. A well-documented route is adsorption of contaminants to the yeast cell wall. The mechanisms of *S. cerevisiae* var. *boulardii* in food detoxification is described in Figure 2. This pathway contributes substantially to the removal of mycotoxins and some PTE. The cell wall’s mannans and β-1,3/β-1,6 glucans overlaying chitin expose phosphate/carboxyl groups and hydrophobic domains that engage planar aromatics and coordinate cations [70,79]. Binding of mycotoxins to yeast cell walls lowers absorption and toxicity [13,68]. Illustrative findings include high binding of total aflatoxins by viable *S. cerevisiae* (74.7%) and removal of ochratoxin A by *S. cerevisiae* var. *boulardii* up to 44% under simulated gastrointestinal conditions [80,81]. In reconstituted milk, *S. cerevisiae* var. *boulardii* achieved 96.88% removal of AFM_1_ at 37 °C, outperforming other probiotic strains [82]. Because adsorption depends on wall integrity and composition [13], binding can be reversible; toxins may desorb if not eliminated promptly, such as through regular bowel movements [83]. For industrial applications, this indicates that adsorption alone may require stabilization approaches or efficient separation steps. To improve stability and enable reuse, disrupted cell-wall fractions of *S. cerevisiae* and *L. rhamnosus* were immobilized onto nano-silica and subsequently entrapped within alginate beads. This combined disruption and immobilization significantly enhanced AFM_1_ removal, with reductions ranging from 53% for free cell-wall fractions (15 min) to 87% for alginate-entrapped adsorbents (24 h). Moreover, the immobilized beads were reusable, maintaining around 85% adsorption efficacy [84].

Yeast cell walls present functional groups that can bind metal ions and support biosorption [85]. Using yeast biomass as a sorbent is attractive economically because it can be sourced as an industrial byproduct [86]. Performance, however, appears strain specific. *Rhodotorula mucilaginosa* shows high tolerance and removal for Hg, Cu, and Pb, whereas planktonic *S. boulardii* tends to tolerate only Pb and forms weak biofilms—a trait that can limit metal capture [87]. These contrasts underscore a knowledge gap and argue against a one size fits all approach [88]. A finer grained understanding of cell wall–metal interactions could guide strategies to strengthen binding, from genetic tuning to combining *S. cerevisiae* var. *boulardii* with complementary binders [89]. Beyond adsorption, *S. cerevisiae* var. *boulardii* secretes enzymes that directly inactivate toxins. The 54 kDa serine protease (*ysp3*) hydrolyzes and neutralizes Clostridioides difficile toxins A and B [37,90], and a 63 kDa phosphatase (*pho8*) can inactivate *E. coli* endotoxin [91]. In models of DON exposure, *S. cerevisiae* var. *boulardii* reversed toxin induced transcriptomic changes and attenuated NF κB and p38 MAPK signaling [92]. Taken together, these findings indicate protection that extends beyond toxin removal to restoration of host pathways, offering a more complete detoxification strategy than adsorption alone [75,93]. The mechanistic diversity of *S. cerevisiae* var. *boulardii* aligns with different application formats: adsorption and biosorption support the use of inactivated cells or isolated cell-wall fractions in foods, whereas enzymatic and host-modulatory activities favor co-ingestion of viable cells. Reported process parameters such as dose, contact time, and pH or formulation correlate with the highest contaminant removals in dairy matrices (e.g., AFM_1_ reductions of up to 96.88% at ~37 °C within 90 min) and with encapsulated preparations in simulated gastrointestinal tract systems for AFB_1_, as discussed previously [82] and summarized in Table 2. Because adsorption-based binding is reversible, industrial applications should incorporate stabilization strategies (e.g., immobilization) or efficient separation steps to minimize desorption.

### 3.2. Applications of Saccharomyces cerevisiae var. boulardii for Detoxification of Food Contaminants

The studies to date span multiple contaminants and matrices. Table 2 summarizes conditions, doses, and outcomes reported for *S. cerevisiae* var. *boulardii*. The detoxification of chemical contaminants in food depends on several factors, including contaminant concentration, incubation time, and pH. However, the limited number of studies to date and the significant heterogeneity in methodologies and outcome measurements make direct comparison challenging. Contaminant concentration is an important factor in the ability of *S. cerevisiae* var. *boulardii* strains to bind toxic compounds. Khadivi et al. [22] showed that a mix of *L. rhamnosus*, *L. plantarum*, and *S. cerevisiae* var. *boulardii* (10^7^ CFU/mL) achieved a 100% adsorption rate of AFM_1_ at an initial concentration of 0.75 ng/mL, while the adsorption rate was 93% with an initial concentration of 0.5 ng/mL [23]. Martínez et al. [22] observed 25% of AFM_1_ (34.89 ng/mL) adsorption by *S. cerevisiae* var. *boulardii* at cell concentration of 10^7^ CFU/mL.

Incubation time also plays an important role in detoxification efficacy. Silva et al. [94] used the yeasts *S. cerevisiae* var. *boulardii* and *S. cerevisiae* and the bacterium *L. delbrueckii* to investigate the reduction of AFB_1_ produced by *A. parasiticus*. The probiotic strain *S. cerevisiae* var. *boulardii* (10^7^ CFU/mL) adsorbed 65.8% of the AFB_1_ produced by the fungus during the 7-day incubation period [94]. Rezasoltani et al. [82] evaluated *S. cerevisiae* var. *boulardii*, *L. casei*, or *L. acidophilus* for removal of AFM_1_ at two different incubation times (30 and 90 min). They observed that among the three strains used, the yeast *S. cerevisiae* var. *boulardii* achieved the highest percentage of AFM_1_ removal, reaching 96.8% at a cell concentration of 10^9^ CFU/mL with 90 min of incubation. Under the same conditions, the yeast reached 91.5% under an incubation time of 30 min [82]. pH is also an important factor that can influence the effectiveness of probiotic strains in absorbing toxic compounds. Pereyra et al. during a gastrointestinal simulation, analyzed the adsorption capacity of AFB_1_ by *S. cerevisiae* var. *boulardii* grown in different culture media. After simulation, with an alkaline condition (pH = 8), *S. cerevisiae* var. *boulardii* produced in a medium of soluble extract of dried distillers’ grains was able to bind 5.72 μg/g of AFB_1_ [95]. At neutral environment (pH = 7), *S. cerevisiae* var. *boulardii* obtained an AFB_1_ adsorption capacity of 86.7% when coated with whey protein concentrate and lyophilized [96].

While studies do not directly evaluate detoxification in foods; therefore, in vitro and in vivo findings are included only briefly to illustrate the biological relevance of adsorption mechanisms. In vitro digestive-simulation studies indicate that *S. cerevisiae* var. *boulardii* can bind DON and AFB_1_, reducing their cytotoxicity, consistent with adsorption behavior observed in food-matrix studies [96,97]. Animal studies demonstrate that ingestion of *S. cerevisiae* var. *boulardii* mitigates downstream physiological effects of DON exposure, supporting the biological relevance of its adsorption capacity [92,98]. Additional rodent studies show attenuation of toxic effects from phthalates, bisphenol A, and pesticides when probiotics, including *S. cerevisiae* var. *boulardii*, are administered [54,99,100]. Probiotic mixtures containing *S. cerevisiae* var. *boulardii* have also shown protective effects in rodent models of arsenic exposure [101,102,103]. Reported efficacy varies with strain/phenotype (including flocculation and wall composition), contact time (minutes–days), dose (1 × 10^7^–1 × 10^9^ CFU/mL), contaminant load, and matrix. Many studies use model systems and do not include desorption tests, realistic residue levels, or sensory endpoints, and strain identifiers are sometimes missing. Since adsorption is inherently reversible, desorption may occur during food processing, storage, or digestion, which represents a critical limitation that must be assessed for industrial applications. Adsorption is often rapid yet reversible, and stability under processing and gastrointestinal-like conditions remains incompletely characterized. To aid comparability and translation, future studies should report the strain ID, dose (log CFU/mL or biomass g/L), contact time, pH, temperature, matrix composition, contaminant level (ng/mL or mg/kg), and outcomes as percent removal (or binding capacity with units), with mass balance where possible.

## 4. Future Perspectives and Research Directions

### 4.1. Enhancing Efficacy Through Genetic and Metabolic Engineering

Genetic and metabolic engineering now make it possible to rationally design improved *S. cerevisiae* var. *boulardii* strains. While numerous such strategies have been developed, their relevance to food-detoxification applications depends on whether they improve contaminant binding, stability, or performance in food matrices. A recent work adapting CRISPR-Cas9 to this yeast opened the door to targeted strain improvement [104,105,106], which could be applied to strengthen cell-wall binding sites or reduce desorption in food systems. A clear priority is to increase binding affinity and stability toward mycotoxins and PTE. One route is to engineer flocculins and other cell-wall adhesins so that interactions with target contaminants are stronger and ideally less reversible [107]. Such modifications are directly relevant to improving adsorption efficiency in food-based detoxification processes. For example, an *S. cerevisiae* var. *boulardii* strain expressing a fibronectin-targeting ligand showed enhanced adhesion to inflamed mucosa [108]. However, its relevance lies only in illustrating how targeted ligand expression could also be directed toward contaminant motifs relevant to foods. In parallel, engineering can amplify endogenous detoxification enzymes (e.g., proteases, phosphatases) or introduce novel catabolic activities to broaden the range of degradable compounds [109,110]. Applying these enzymatic strategies to foodborne contaminants would broaden the yeast’s capacity beyond adsorption alone. Feasibility has already been demonstrated by expressing human lysozyme in *S. cerevisiae* var. *boulardii* for gut health applications [106], although this is not food-specific and should be interpreted only as evidence that foreign proteins can be expressed successfully. From a regulatory and safety perspective, strains that avoid antibiotic markers are desirable [105]. Auxotrophic systems such as uracil auxotrophy allow selection and maintenance without antibiotics and remain a practical framework for industrial and clinical use [110,111]. Hence, such systems should be considered primarily insofar as they support the safe use of engineered strains for food-detoxification purposes.

### 4.2. Elucidating Novel Mechanisms with Multi-Omics Technologies

Systems biology tools can move the field beyond descriptive findings to mechanism. Metabolomics, proteomics, and transcriptomics together can reveal the molecular basis of *S. cerevisiae* var. *boulardii*’s actions [112]. When applied to food matrices, these approaches can identify specific metabolites, cell-wall components and or proteins that directly govern contaminant binding or influence adsorption stability. Metabolomics, for instance, can profile the exometabolome and uncover bioactive molecules that extend beyond known enzymes and binding proteins [113]. These analyses may clarify whether secreted or surface-associated metabolites enhance contaminant sequestration in foods. Non-targeted analyses have already detected metabolites such as phenyllactic acid and 2-hydroxyisocaproic acid in *S. cerevisiae* var. *boulardii*, absent from *S. cerevisiae*, that may underpin distinct antioxidant and antimicrobial properties [41]. Only those metabolites affecting contaminant interactions in food systems are relevant to the detoxification context. Proteomics and transcriptomics, in turn, can map secreted proteins, cell-wall components, and host–microbe interactions across food matrices and tissues [114]. These valuable tools can be used to identify the binding motifs, stress-response pathways, and adsorption-associated structures that determine strain performance in foods. As example, transcriptomic data indicate that *S. cerevisiae* var. *boulardii* can reverse the DON-induced shift in intestinal gene expression and dampen inflammatory signaling (NF-κB, p38 MAPK) [92]. Though this reflects post-ingestion physiological protection rather than food-matrix detoxification and is therefore only indirectly relevant. Future omics work should instead focus on how the yeast behaves within food environments, which components mediate contaminant binding, and how processing conditions influence these mechanisms.

### 4.3. Synergistic Biocontrol Communities for Scalable Food Detoxification

Progress will likely require a change from single-strain solutions to multi-species consortia. Combining *S. cerevisiae* var. *boulardii* with LAB can generate complementary modes of action and improve functional stability across diverse food matrices [115]. Studies already demonstrate LAB–yeast synergy, including enhanced growth, survival, and technological performance in food systems [21,116]. These interactions may also strengthen adsorption performance via co-aggregation or complementary binding activities. Further robustness and detoxification capacity can be achieved by pairing *S. boulardii* with non-living components such as prebiotics or postbiotics, especially if these enhance adsorption stability or reduce desorption during food processing or storage [115]. From a translational perspective, major bottlenecks lie in production, stabilization, and delivery. Cost-effective, large-scale fermentation requires optimization for biomass yield and retention of functional traits, but only those that capably affect contaminant binding and stability in the food matrices of interest. Stabilization methods more economical than freeze-drying should be prioritized [117], provided they preserve adsorption capacity and minimize post-processing desorption. Integrating these elements, as consortium design, bioprocess optimization, and practical delivery formats, will be essential to developing *S. cerevisiae* var. boulardii-based detoxification systems that are feasible for large-scale food applications [118,119]. Immobilization or entrapment of yeast cell-wall fractions on nano-silica or alginate matrices is one promising approach, as it reduces desorption, enables adsorbent reuse, and maintains high AFM_1_ removal efficiencies [84].

Probiotic candidates intended for food applications must meet GRAS (U.S.) or QPS (EU) criteria [120], exhibit minimal risk of antimicrobial-resistance gene transfer [121], and demonstrate product- and gastrointestinal-stability during scalable, genetically stable manufacturing processes [122]. For food-detoxification uses, the primary considerations include contaminant-binding stability, safety of the yeast preparation, and its impact on food quality. *Saccharomyces cerevisiae* holds GRAS and QPS status; *S. cerevisiae* var. *boulardii* falls within this species, but product- and strain-specific safety evaluations remain necessary when proposed as a decontamination aid. In food matrices, viable yeasts may cause spoilage through gas and ethanol production or alterations to texture and flavor, making heat-inactivated cells or purified cell-wall fractions more suitable for pre-consumption detoxification. For co-ingestion applications, rare cases of fungemia have been reported in severely immunocompromised individuals; thus, GMP/HACCP controls, appropriate strain selection, and clear labeling are required to mitigate risk. Regulatory approval typically involves submission of a GRAS notice in the U.S. or a QPS-based dossier in the EU specifying intended use, safety characterization, controls on residual activity, and potential sensory impacts. Within the context of food detoxification, regulatory considerations should emphasize functional stability, desorption risk, and performance consistency under real processing conditions.

## 5. Conclusions

Chronic dietary exposure to chemical contaminants contributes to a broad spectrum of toxic effects. Although the current evidence base is modest, studies on the probiotic yeast *S. cerevisiae* var. *boulardii* has potential for biological detoxification across mycotoxins, pesticides, PTE, and packaging migrants such as BPA and phthalates. Performance is shaped by contaminant load, contact time, pH, strain and dose, formulation (e.g., encapsulation), and properties of the food matrix. Adsorption predominates, with complementary contributions from enzymatic detoxification, host modulatory effects, anti-virulence activity, and competitive exclusion. Two persistent obstacles are the reversibility of binding and methodological heterogeneity across studies. Priority work should now focus on: (i) standardized protocols across matrices and contaminant classes; (ii) disentangling adsorption versus degradation and stabilizing binding; (iii) dose–response and kinetic data under realistic processing and storage conditions; (iv) sensory, quality, and safety assessments; (v) scalable production and delivery formats (viable, inactivated, or cell wall fractions); and (vi) clear regulatory pathways for food applications. Progress on these fronts will help move *S. cerevisiae* var. *boulardii*-based detoxification from promise to practice and reduce the burden of foodborne chemical hazards.

## Figures and Tables

**Table 1:** Physiological and genetic distinctions between *Saccharomyces cerevisiae* var. *boulardii* and *S. cerevisiae*.

Trait	*Saccharomyces cerevisiae* var. *boulardii*	*Saccharomyces cerevisiae*
Growth temperature	Optimal at 37 °C	Optimal at 30 °C
pH and bile salt tolerance	Enhanced survival under simulated gastric (pH ≤ 2) and bile stress	Strong acid tolerance in fermentation range (pH 3–3.5); lower survival than boulardii under combined low-pH and bile stress
Sporulation	Does not form ascospores	Forms ascospores
Galactose metabolism	Cannot digest galactose	Digests galactose
Genetic features	Extra copies of *FLO* genes (flocculins); mutations in MATa locus; altered copy numbers in stress-response genes	Lower *FLO* copy number; functional MATa locus (sporulation); standard copy numbers

Adapted from [28,29,30].

**Table 2:** Applications of *Saccharomyces cerevisiae* var. *boulardii* in for detoxification of food contaminants.

Contaminant	Initial Concentration	Adsorbent Dose	Matrix/Model	Strain	Contact Time	Outcome (Removal%, Binding, or Protective Effect)	Ref.
AFB_1_	0.8–0.88 ng/mL	8.0 log_10_ CFU/mL	Peanuts	*S. cerevisiae* var. *boulardii*-17	7 days	Removal: 65.8%	[94]
AFB_1_	150 ng/mL	7.0 log_10_ CFU/mL	Simulated GIT	*S. cerevisiae* var. *boulardii* RC009	40 min	Binding capacity: 3.77 μg/g	[95]
AFB_1_	50 ng/mL	9.0 log_10_ CFU/mL	Simulated GIT	*S. cerevisiae* var. *boulardii* RC009	4 h	Removal: 79.5–86.7% (encapsulated)/40–33% (formulation-dependent)	[96]
AFM_1_	0.75 ng/mL	7.0–9.0 log_10_ CFU/mL	Reconstituted low-fat milk powder	NS	90 min	Removal: 45.93%/96.88%	[82]
AFM_1_	50 ng/mL	7.0 log_10_ CFU/mL	Milk	*S. cerevisiae* var. *boulardii* RC009	60 min	Removal: 25%	[22]
AFM_1_	0.75 ng/mL	7.0 and 9.0 log_10_ CFU/mL	Low-fat milk	NS	90 min	Removal: 90.66%/75.42%	[23]
DON	10 μM	5.5 log_10_ CFU/mL	Male piglet jejunal explants	*S. cerevisiae* var. *boulardii* CNCM I-1079	30 min or 4 h	Protective effect: Pathway modulation	[92]
DON	NA	NA	Porcine alveolar macrophage cells (in vitro)	NS	45 min	Protection vs. apoptosis/necrosis; ↓ IL-6, TNF-α, IL-1β	[97]
DON	2.82 mg/kg feed	4 × 10^9^ CFU/kg feed	Male piglets (intestine, liver, kidney)	*S. cerevisiae* var. *boulardii* CNCM I-1079	28 days	Histological restoration; metabolic normalization	[98]
Acetamiprid	12.4 mg/kg	9.0 log_10_ CFU/mL	Wistar rats (testis)	NS	90 days	Protective effect vs. tissue degeneration	[99]
Imidacloprid	5.7 mg/kg	NS	Wistar rats	NS	90 days	Protective effect vs. tissue degeneration	[99]
DEHP (phthalate)	Corn oil: 1.0 mL/kg body weight	8.8 × 10^8^ CFU/kg/day	Wistar rats (organs)	Mix including *S. cerevisiae* var. *boulardii*	28 days	Protection vs. hepato/renal toxicity	[54]
DEHP, DBP, BPA (mix, in vivo)	DEHP/DBP 50; BPA 25 mg/kg/day	8.8 log_10_ CFU/mL	Male albino rats	Mix including *S. cerevisiae* var. *boulardii*	28 days	Reduced oxidative/pancreatic damage	[100]
DEHP, DBP, BPA (mix, in vitro)	Same doses	9.4 log_10_ CFU/mL	In vitro	*S. cerevisiae* var. *boulardii* + *Lactobacillus* spp.	4 h	Binding: 29.25% (DEHP), 29.04% (DBP), 41.75% (BPA)	[54]
As	1.0 mg/100 g bw	Probiotic mix (100 mg)	Wistar rats (blood, uterus, ovaries)	Mix incluing *S. cerevisiae* var. *boulardii*	16 days	Protection; serum B12 restoration; ↓ NF-κB activation	[101]

AFB_1_: aflatoxin B_1_; AFM_1_: aflatoxin M_1_; As: arsenic; DON: deoxynivalenol; DEHP: bis(2-ethylhexyl) phthalate; DBP: dibutyl phthalate; BPA: bisphenol A; CFU/mL: colony-forming unit per milliliter; GIT: gastrointestinal tract; NA: not applicable; NS: not specified; ↓: reduced.

## Data Availability

No new data were created or analyzed in this study.
